# A 12-year comparison of students’ perspectives on diversity at a Jesuit Medical School

**DOI:** 10.3402/meo.v19.23401

**Published:** 2014-02-27

**Authors:** Imran Mujawar, Matt Sabatino, Stephen Ray Mitchell, Benjamin Walker, Peggy Weissinger, Michael Plankey

**Affiliations:** 1 Department of Medicine, Georgetown University Medical Center, Washington, DC, USA; 2 Office of the Dean for Medical Education, School of Medicine, Georgetown University, Washington, DC, USA

**Keywords:** medical students, diversity, curriculum, equality, survey, identity

## Abstract

**Background:**

Many studies have assessed perspectives of medical students toward institutional diversity, but few of them have attempted to map changes in diversity climate over time.

**Objective:**

This study aims to investigate changes in diversity climate at a Jesuit medical institution over a 12-year period.

**Methods:**

In 1999, 334 medical students completed an anonymous self-administered online survey, and 12 years later, 406 students completed a comparable survey in 2011. Chi-square tests assessed the differences in percent responses to questions of the two surveys, related to three identities: gender, race, and sexual orientation.

**Results:**

The 1999 versus 2011 samples were 46% versus 49% female, 61% versus 61% Caucasian, and 41% vs. 39% aged 25 years or older. Findings suggested improvements in medical students’ perceptions surrounding equality ‘in general’ across the three identities (*p*<0.001); ‘in the practice of medicine’ based on gender (*p*<0.001), race/ethnicity (*p*=0.60), and sexual orientation (*p*=0.43); as well as in the medical school curriculum, including course text content, professor’s delivery and student–faculty interaction (*p*<0.001) across the three identities. There was a statistically significant decrease in experienced or witnessed events related to gender bias (*p*<0.001) from 1999 to 2011; however, reported events of bias based on race/ethnicity (*p*=0.69) and sexual orientation (*p*=0.58) only showed small decreases.

**Conclusions:**

It may be postulated that the improvement in students’ self-perceptions of equality and diversity over the past 12 years may have been influenced by a generational acceptance of cultural diversity and, the inclusion of diversity training courses within the medical curriculum. Diversity training related to race and sexual orientation should be expanded, including a follow-up survey to assess the effectiveness of any intervention.

## Introduction

The Liaison Committee on Medical Education (LCME) defines an appropriate institutional setting for medical education as an ‘environment characterized by, and supportive of, diversity and inclusion’ (1). Diversity training in medical education is not only essential for academic excellence during medical training, but has also shown to provide far-reaching benefits later on in medical practice, including health disparity reduction, better physician–patient interaction, as well as enhanced patient adherence, satisfaction, and clinical outcomes (2, 3).

Institutional leadership can help define this essential atmosphere of cultural competency with ‘creation of curricula and environments’ that supports critical dialogue on the potentially contentious issues of gender, race/ethnicity, and sexual orientation (4). Therefore, it is important to assess cultural climate related to diversity at medical schools for augmentation and benchmarking the system (5).

There have been many studies gauging perceptions and attitudes of medical students toward diversity and cultural competency, with or without interventions (6–9). Very few longitudinal studies have attempted to map changes in cultural climate and institutional discourse on diversity over time. A longitudinal qualitative study evaluating gendered encounters in medical education revealed that third-year female medical students easily acclimatized to inappropriate behavior from male patients, but not toward unprofessional behavior of male supervisors (10). Another study with a 4-year follow-up survey administered to 185 lesbian, gay, and bisexual (LGB) medical students across 92 medical schools in the United States, found that LGB medical students’ needs were being increasingly met, but student–faculty liaisons and more support groups were needed; LGB medical students also felt there was a paucity of exposure to non-pejorative descriptions of LGB patients, and a need for LGB patient care being taught more widely (11).

To the best of our knowledge, this article documents the first longitudinal study assessing changes in diversity climate at a Jesuit medical institution, which espouses cultural competency through its teaching of *Cura Personalis*, an Ignatian principle adapted for healthcare. Translated as ‘care of the whole person’, *Cura Personalis* advocates individualized attention to other’s needs, ‘distinct respect for unique circumstances’, and a suitable appreciation for ‘singular gifts and insights’ (12). The study compares students’ responses to surveys related to gender, race, and sexual orientation-based diversity and bias, which were conducted in 1999 and again in 2011.

## Methods

### Participants

In 1999, 334 first- and second-year medical and post-baccalaureate physiology graduate students at a private, Jesuit medical school in the US completed an anonymous self-administered online survey. Twelve years later, 406 first- and second-year medical and post-baccalaureate physiology graduate students completed a comparable survey from January to April, 2011.

### Data collection

The two cohorts were compared, estimating the differences in percent responses to questions of the two surveys. The study focused on responses to matching questions in both surveys on gender, race/ethnicity, and sexual orientation-based equality (Table 1) related to: 1) level of importance attributed to equality in general; 2) perceptions of equality in the practice of medicine; 3) students’ assessment of equality based on gender, race, and sexual orientation in the curriculum; and 4) witnessing or experiencing gender, race/ethnicity, and sexual orientation bias.

**Table 1:** Survey questions in both 1999 and 2011 studies

Theme	Question	Response
Importance of equality in general	1) How important is it to you that men and women are treated equally? 2) How important is to you that people of different races/ethnicities are treated equally? 3) How important is to you that people of different sexual orientations are treated equally?	Not at all, Slightly, Moderately, A lot, Extremely
Equality in the practice of medicine	4) Do you believe women and men are treated equally in the practice of medicine? 5) Do you believe people of different race/ethnicity are treated equally in the practice of medicine? 6) Do you believe people with different sexual orientations are treated equally in the practice of medicine?	Yes, No
Equality in medical curriculum	7) At the medical school, do you believe that men and women are treated equally in: 1) Course texts; 2) Professor’s delivery; 3) Student–Faculty interaction? 8) At the medical school, do you believe that people of different race/ethnicity are treated equally in: 1) Course texts; 2) Professor’s delivery; 3) Student–Faculty interaction? 9) At the medical school, do you believe that people with different sexual orientations are treated equally in: 1) Course texts; 2) Professor’s delivery; 3) Student–Faculty interaction?	Yes, No
Witnessing or experiencing bias at the medical school	10) Have you personally witnessed or experienced bias based on gender at the medical school? 11) Have you personally witnessed or experienced bias based on race/ethnicity at the medical school? 12) Have you personally witnessed or experienced bias based on sexual orientation at the medical school?	Yes, No

### Data analysis

For each identity, we calculated the percentage of respondents choosing each category and estimated differences between the percent responses for 1999 and 2011. Chi-square tests assessed whether there were any significant differences in the responses between the two survey years. Statistical significance was evaluated at the 0.05 level. The data were analyzed using SAS version 9.3 (SAS Institute Inc., USA).

## Results

The 1999 vs. 2011 sample demographic compositions were as follows: 46% (153/334) vs. 49% (199/406) female, 61% (204/334) vs. 61% (247/406) Caucasian, and 41% (137/334) vs. 38% (156/406) were older than 25 years. The two samples were not significantly different with respect to gender, age, student status, and race (Table 2).

**Table 2:** Demographic data for medical students participating in the 1999 and 2011 studies

Variable	Study 1999 (*N*=334) *n* (%)	Study 2011 (*N*=406) *n* (%)	*p*
Gender			0.198
Male	178 (53.3)	207 (51.0)	
Female	153 (45.8)	199 (49.0)	
Missing data	3 (0.9)	0	
Age			0.094
≤22 years	50 (15.0)	46 (11.3)	
23–24 years	147 (44.0)	204 (50.2)	
25–26 years	74 (22.1)	99 (24.4)	
27–29 years	41 (12.3)	43 (10.6)	
≥30 years	22 (6.6)	14 (3.5)	
Student status			0.281
1st Year	123 (36.8)	158 (38.9)	
2nd Year	137 (41.0)	139 (34.2)	
GEMS	11 (3.3)	17 (4.2)	
Grads	63 (18.9)	90 (22.2)	
Missing data	0	2 (0.5)	
Race			0.381
Black	25 (7.5)	25 (6.2)	
Asian	60 (18.0)	81 (20.0)	
White	204 (61.1)	247 (60.8)	
Latino	14 (4.1)	17 (4.1)	
Indian	14 (4.2)	10 (2.5)	
Middle Eastern	5 (1.5)	15 (3.7)	
Native American	2 (0.6)	1 (0.2)	
Missing data	10 (3.0)	10 (2.5)	

**Fig. 1:**
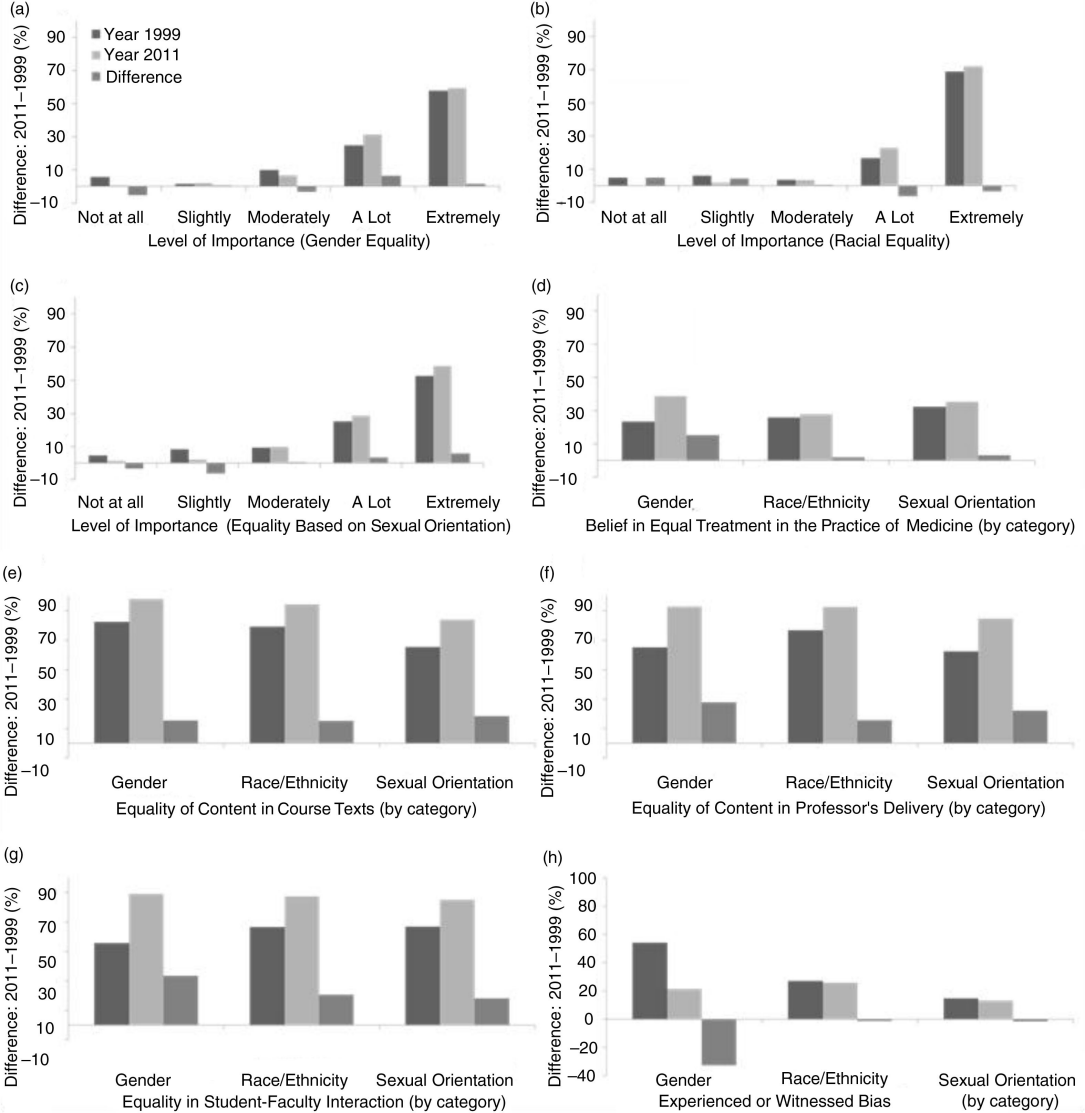
Response comparisons between the 1999 and 2011 surveys.

Students in the 2011 survey, as compared to students in the 1999 survey, placed significantly greater value on the importance of equality in general based on gender (24.8% vs. 31.3% gave ‘a lot’ of importance, *p*<0.001); race/ethnicity (16.7% vs. 23% gave ‘a lot’ of importance, *p*<0.001); and sexual orientation (52.5% vs. 58.4% responded ‘extremely’ important, *p* <0.001) (Fig. 1a–c).

Students in 2011 were more likely than students in 1999 to ‘believe in equality in the practice of medicine’, based on gender (15.3%, *p*<0.001), race (1.8%, *p*=0.60), and sexual orientation (3.1%, *p*=0.43), with gender equality demonstrating a statistically significant improvement (Fig. 1d).

Researchers noted a positive increase from 1999 to 2011 in the students’ perception of equality based on gender, race, and sexual orientation at the medical school in course text content, professor’s delivery and student–faculty interaction (*p*<0.001); gender equality in student–faculty interaction and professor’s delivery demonstrated the greatest positive increase of 33.5 and 27.9%, respectively (Fig. 1e–g).

There was a statistically significant decrease in experienced or witnessed events related to gender bias (54.2% vs. 21.4%, *p*<0.001) from 1999 to 2011 (Fig. 1f); however, reported events of bias based on race/ethnicity (27.2% vs. 25.9%, *p*=0.69) and sexual orientation (14.6% vs. 13.2%, *p*=0.58) only showed small decreases that were not statistically significant (Fig. 1f).

## Discussion

We found improvements in medical students’ perceptions surrounding equality in general, in the practice of medicine, and in the medical curriculum. We also found improvement in the decreasing percentage of students who have witnessed or experienced negative bias based on gender, race/ethnicity and sexual orientation, albeit those for race/ethnicity and sexual orientation were not statistically significant.

In this study, students’ perceptions and attitudes toward gender equality showed a substantial positive increase, reflecting the shift in focus of gender issues in academic medicine from mitigating discrimination and sexual harassment (SH) to facilitating an environment conducive for excellence in medicine. Three previous questionnaire-based studies demonstrated that gender-based discrimination (GD) and SH were prevalent in undergraduate medical education as well as among medical school faculty (13–15). A 2013 study measuring the key aspects of academic medicine culture noted that though male and female medical academicians were equally engaged in their work and had similar professional aspirations, medical institutions have failed to provide an environment supporting and accepting of women in medicine (14). Another recent study surveyed 4,578 full-time faculty from 26 representative US medical schools and noted that gender was not predictive of intentions for leaving academic medicine (15). Looking at perspectives in society-at-large, a 2013 Gallup poll indicated that 85% of women did not perceive gender bias in promotions at work (16). The current study and others would support the fact that, gender biases, do still exist within the faculty of the biological and physical sciences, but on a more subtle level (17).

US national-level surveys indicate that small shifts in attitudes and perspectives on racial equality have occurred over the past decade, reflecting results of our study. A 2013 Pew Research survey, assessing diversity and inclusion within society-at-large, indicated that 35% of Blacks, 20% of Hispanics, and 10% of Whites say that they have experienced discrimination because of their race and ethnicity over the past year (18). Moreover, the Pew survey noted that ‘while 45% of Americans say the country has made ‘a lot’ of progress in the past 50 years toward racial equality, about half the American public says ‘a lot’ more needs to be done’.

For changes in diversity regarding sexual orientation, a 2013 *Pew* survey indicated that 92% of America’s LGBT (Lesbian, Gay, Bisexual, and Transgender) adults say ‘society has become more accepting of them in the past decade’ and an equal number expect it to ‘grow even more accepting in the decade ahead’ (19). While the acceptance of homosexuality over the past decade increased from 40% in 2001 to 56% in 2011 (20), 63% of Americans describe discrimination based on sexual orientation as a serious problem (21). Our results showed only a 1.4% decrease in self-reported events of ever witnessing or experiencing negative bias based sexual orientation at the medical school over the past decade, even though Washington, DC has the highest LGBT percentage in the United States (22). This demonstrates the prevalence of how implicit, or unconscious, biases against certain groups can influence behavior, even when measures of explicit beliefs show no group biases (23–25).

An online survey administered nationally to 427 LGBT physicians revealed that LGBT educational content has not been sufficiently included in formal medical school curriculum to bring about an alteration in the ‘heterosexist and gender normative practices’ in medical practice (26). Moreover, there seems to be a paucity of cross-cultural medical training during clinical rotations in the United States (2). Another study evaluating sexuality education focused on North American medical students, noted that education material on sexual minority groups is scant or absent in most curriculum (27). Meanwhile, a university in California assessed the impact of an LGBT health curriculum on second-year medical students by administering matched questionnaires eliciting knowledge, attitudes, and beliefs of students about LGBT health issues before and after completion of the course. The study demonstrated that a ‘simple curricular intervention’ could produce significant short-term changes in a small number of survey items (7). Sustaining those changes may require a longer-term plan.

While our results indicate positive changes in student perspectives on equality based on gender, race, and sexual orientation, they could possibly be explained by the shifting societal perspectives on gender and racial equality. Our results could also be the result of a concerted effort by the medical school. Guided by the Jesuit core value of *Cura Personalis*, to create and increase a diverse medical school environment, the Medical School Executive Faculty updated the school of medicine diversity statement:

The University was founded on the principle that serious and sustained discourse among people of different faiths, cultures and beliefs promotes intellectual, ethical and spiritual understanding. Consistent with this principle, the School of Medicine strives to ensure that its students become respectful physicians who embrace all dimensions of diversity in a learning environment that understands and includes the varied health care needs and growing diversity of the populations we serve. (28)

In keeping with this stated mission, the School of Medicine has implemented outreach programs in order to facilitate the diversity of the student body. In addition, our university has initiated periodic campus-wide initiatives to promote a respectful campus community to increase an environment of anti-harassment, promote diversity and equality, and raise awareness of diversity issues on campus. These efforts have been combined with an integration of diversity topics into the curriculum to provide students with the tools required to practice healthcare in an increasingly diverse healthcare arena.

Our study was limited to assessing pre-clinical education only and therefore cannot be extrapolated to clerkship rotations where a higher prevalence of bias may be expected due to increased clinician–student–patient interactions. Moreover, any underreporting of responses to these sensitive questions would underestimate any differences particularly among those who are highly vulnerable to experiencing or witnessing bias.

We postulate that the improvement in students’ self-perceptions of equality and diversity at the medical school over the past 12 years may have been influenced by two primary factors: 1) a generational acceptance of cultural diversity as reflected in the society-at-large facilitated by the use of the Internet and social networks; and 2) the inclusion of diversity training and health disparities courses within the medical curriculum. Diversity training related to race and sexual orientation should be expanded, including a follow-up survey to assess the effectiveness of any intervention.
